# Peripartum Cardiomyopathy During Puerperium Co-existing with Pre-eclampsia and Complicated by Acute Renal Failure: A Case Report

**DOI:** 10.7759/cureus.6319

**Published:** 2019-12-07

**Authors:** Arshia Akbar, Hafiz Muhammad Ata ur-Rehman, Asim Tameez Ud Din, Aimen Malik

**Affiliations:** 1 Internal Medicine, Rawalpindi Medical College, Rawalpindi, PAK; 2 Internal Medicine, Shifa International Hospital, Islamabad, PAK; 3 Internal Medicine, Rawalpindi Medical University, Rawalpindi, PAK; 4 Internal Medicine, College of Physicians and Surgeons Pakistan, Islamabad, PAK

**Keywords:** peripartum cardiomyopathy, severe left ventricular dysfunction, pregnancy

## Abstract

We report a case of peripartum cardiomyopathy (PPCM), which presented with antenatal pre-eclampsia complicated by acute kidney injury (AKI). A 25-year-old patient in her 27^th^ week of gestation presented with high blood pressure. She was later diagnosed with PPCM, which was complicated by AKI. Our case report indicated PPCM presentation during the prepartum period, which is a rare entity. On her fifth day of admission, our patient had spontaneous expulsion of her neonate, who was found to be dead on antenatal ultrasound.

## Introduction

Peripartum cardiomyopathy (PPCM) is a rare form of heart failure that can present with mild or severe symptoms [1]. The worldwide incidence is variable with the highest rate in Nigeria (one in 102 deliveries), and in African Americans, indicating that it is more prevalent among the black race [2]. The onset is usually in the last month of pregnancy and up to five months post-partum [3]. The most common etiology of this condition is idiopathic [4]. Although it is difficult to diagnose this rare disease, severe cardiomyopathy is self-revealing, presenting with shortness of breath and swollen feet just after the delivery. The severity of the condition can be determined by the ejection fraction [5]. Here, we present an unusual case of PPCM in previously healthy pregnant women who presented with preeclampsia. This case also demonstrates the rare prepartum presentation of PPCM.

## Case presentation

A 25-year-old Asian woman, gravida 2, para 1, with a history of one spontaneous abortion presented in the medical emergency department (ED) with symptoms of orthopnea, paroxysmal nocturnal dyspnea, and leg swelling for four days.

The patient was doing well until the 27th week of pregnancy when she had high blood pressure for which she went to an outpatient clinic. She was diagnosed with pre-eclampsia and gestational hypertension and was treated with nifedipine oral tablets three times a day (TDS). For the last four days, she had mild respiratory symptoms and gradually increasing leg swelling along with chest pain.

The patient did not have any significant past medical problems or allergies. She reported q miscarriage at first pregnancy, with no elective procedure done. She denied alcohol, smoking, or illicit drug use. There was no cardiac history in the family.

**Figure 1:**
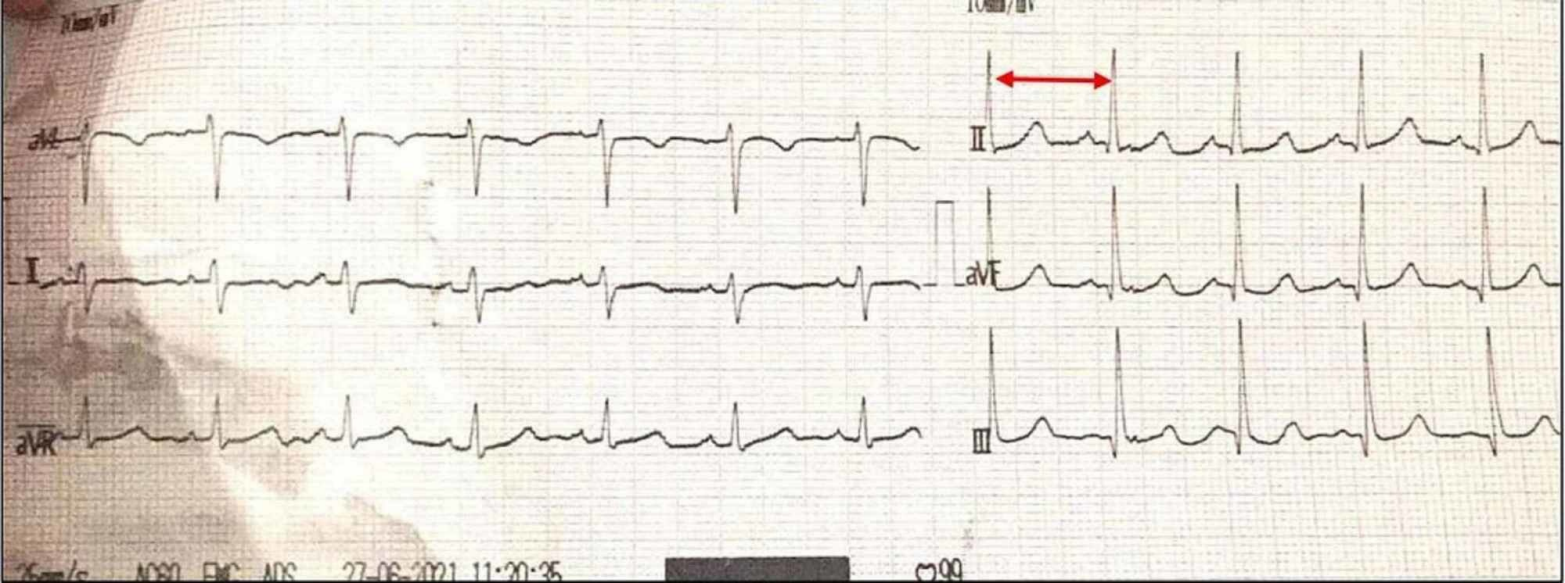
ECG - Sinus Tachycardia ECG: electrocardiogram

**Figure 2:**
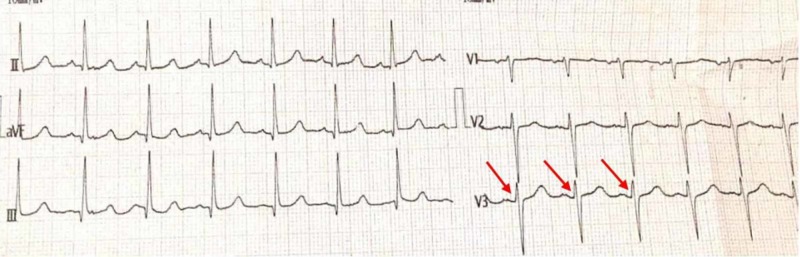
ECG - Poor R-wave Progression ECG: electrocardiogram

On admission, physical examination revealed flushing of the face, with blood pressure (BP) of 150/110, tachycardia, no jugular venous distension, bibasilar crackles with decreased air entry at bases, 3+ pitting edema of legs, reflexes normal, and SpO2 (oxygen saturation) of 71%. Electrocardiography showed sinus tachycardia and poor R-wave progression (Figures 1-2). A chest X-ray showed pulmonary edema with bilateral effusion. Echocardiography showed global hypokinesis with severe left ventricular systolic dysfunction (ejection fraction of 35%). The dimensions recorded for chambers were: left atrium: 36 mm; aortic root: 29 mm, left ventricle end-diastolic (LVED): 53 mm; left ventricle end-systolic (LVES): 43 mm.

The initial laboratory findings are shown in Table 1. A central venous catheter was passed to improve fluid management. Aggressive but careful diuresis with IV (intravenous) furosemide was done. IV nitroglycerin was started to reduce afterload and preload. She did not show much response to diuretics and nitrates and was transferred to the medical intensive care unit (MICU) when her condition deteriorated. She had a Glasgow Coma Scale (GCS) of 8/15 with BP 144/84; pulse rate 115; respiratory rate 35/minute; temperature: afebrile; and SpO2 of 56%. Her arterial blood gases (ABGs) on admission showed mixed metabolic and respiratory acidosis with the values as follows: pH 7.16; partial pressure of carbon dioxide (pCO2) 49.6 mmHg; partial pressure of oxygen (pO2) 35 mmHg; bicarbonate (HCO3) 18 mmol/L; and SpO2 71.6%. Enalapril 5 mg orally twice daily was added to help decrease preload as well as afterload. On the third day of hospitalization, amlodipine 5 mg daily was started for better control of BP. On her fifth day of admission, she had spontaneous expulsion of her neonate, who was found to be dead on antenatal ultrasound.

**Table 1:** Laboratory investigations WBC: white blood cells; pCO2: partial pressure of carbon dioxide; RBC: red blood cells; ALT: alanine aminotransferase; AST: aspartate aminotransferase; ALP: alkaline phosphatase; BUN: blood urea nitrogen; Trop-I: troponin I; CKMB: creatine kinase; CPK: creatine phosphokinase; BNP: brain natriuretic peptide

Laboratory Investigations	Results	Blood Chemistry	Results
WBC	23.4 x 109/L	ALT	404 U/L
Hematocrit	42.90%	AST	982 U/L
PCO2	64.7 mm/Hg	ALP	165 U/L
		BUN	58 mg/dL
Lipid Profile		Creatinine	1.2mg/dL
Total cholesterol	191 mg/dl	Trop-I	1.07 ng/mL
Triglyceride	214 mg/dl	CKMB	56.2 U/L
		CPK	385 U/L
Urinalysis		BNP	4001.9 pg./mL
Proteinuria	1+	Sodium	138 mmol/L
RBC	10–20 /mm3	Potassium	5.1 mmol/L
		Magnesium	4.47 mmol/L
		Phosphorus	8.46 mmol/L
		Albumin	2.42 g/dL

During her hospital stay, her renal function deteriorated and she required three hemodialysis sessions during the stay in MICU. Ultrasound of her abdomen and kidneys showed normal liver and kidney functions.

The patient's repeated radiographs showed improved pulmonary edema, pleural effusion, and a decrease in heart size. She was extubated seven days after admission and IV furosemide was converted to oral.

The patient continued to improve clinically and was discharged home on the 16th day of her admission. Her renal function gradually improved with creatinine levels on a downward trend until normal before discharge.

## Discussion

PPCM is a type of dilated cardiomyopathy of unknown origin. Peripartum cardiomyopathy typically occurs in the first four months after pregnancy, but fewer than 10% of cases also occur prepartum [6]. Our case report indicates PPCM, which presented prepartum. It occurs in one of every 3,000-15,000 pregnancies, with the highest incidence reported in Nigeria [7]. In Pakistan, 3.8 cases are reported per 1000 pregnancies [8].

The etiology of PPCM is unknown. Recent evidence suggests that this disease is a type of myocarditis arising from an infectious, autoimmune, or idiopathic process [9]. The major risk factors for PPCM include advanced maternal age, pre-eclampsia, and multiple gestations. Other cardiovascular risk factors, such as hypertension, diabetes, and obesity, can also attribute to this pathology [10].

Several important physiologic changes occur during the second trimester of pregnancy like blood volume expansion, relative anemia, increased heart rate, metabolic demand, cardiac output, and preload [11]. Women with PPCM usually present with symptoms like dyspnea on exertion, orthopnea, paroxysmal nocturnal dyspnea, and edema of the lower extremities [12]. Less common presentations include symptomatic arrhythmias and arterial thromboembolism [13-14].

Physical examination typically shows evidence of both left-sided congestion (eg, pulmonary rales) and right-sided congestion (raised jugular venous pressure, edema). Electrocardiography shows sinus rhythm, often with non-specific ST-segment or T-wave abnormalities or both [15]. Chest films reveal signs of congestive heart failure, including cardiomegaly, pulmonary edema, and occasionally pleural effusion [16]. An echocardiogram shows normal wall thickness with globular hypokinesis and high intracardiac pressure with low cardiac output [17].

Treatment recommendations are preload reduction and inotropic support. Primary treatment includes bed rest (modest exercise), fluid restriction, and sodium restriction [9]. Diuretics and oral nitrates are used to decrease preload, but diuretic use should be monitored in pregnancy [18]. Over-diuresis during pregnancy could lead to maternal hypotension and uterine hypoperfusion [12].

Controlled studies with β blockers in dilated cardiomyopathy showed improvement in the quality of life, less need for heart transplantation, a significant reduction in the number of hospital readmissions due to a worsening of the heart condition. Results from Cardiac insufficiency BisoproloL Study( CIBIS) proves from previous trials that an increased dose of β blockers in idiopathic dilated cardiomyopathy has a functional benefit [19].

For women who develop PPCM antepartum, a multidisciplinary team comprising obstetrics, anesthesia, and cardiology should individualize patient management based on severe and stabilized acute heart failure [20].

## Conclusions

Peripartum cardiomyopathy is a rare but serious disease that affects women of childbearing age. Diagnosis is mainly based on clinical suspicion and echocardiogram. The management of peripartum cardiomyopathy should aim first at improving heart-failure symptoms through conventional therapies, but it depends on whether the patient is post-partum or still pregnant. Despite the high risk of recurrence, many patients recover within three to six months of disease onset. We present a prepartum case of PPCM, which was complicated by AKI. The patient progressively improved but there was spontaneous expulsion of the neonate on the fifth day of admission who was found dead on ultrasound. The physician should also counsel the patient about future pregnancy and contraception. Further clinical evaluation for targeted therapies (for example, intravenous immunoglobulin, pentoxifylline, and bromocriptine) needs to be done. A large, multicenter, prospective randomized trial is needed to evaluate more about recurrence, prognosis, and therapies of PPCM.
